# Large Scale Comparison of Gene Expression Levels by Microarrays and RNAseq Using TCGA Data

**DOI:** 10.1371/journal.pone.0071462

**Published:** 2013-08-20

**Authors:** Yan Guo, Quanhu Sheng, Jiang Li, Fei Ye, David C. Samuels, Yu Shyr

**Affiliations:** 1 Center for Quantitative Sciences, Vanderbilt University, Nashville, Tennessee, United States of America; 2 Center for Human Genetics Research, Dept. of Molecular Physiology and Biophysics, Vanderbilt University Medical Center, Nashville, Tennessee, United States of America; University of Turin, Italy

## Abstract

RNAseq and microarray methods are frequently used to measure gene expression level. While similar in purpose, there are fundamental differences between the two technologies. Here, we present the largest comparative study between microarray and RNAseq methods to date using The Cancer Genome Atlas (TCGA) data. We found high correlations between expression data obtained from the Affymetrix one-channel microarray and RNAseq (Spearman correlations coefficients of ∼0.8). We also observed that the low abundance genes had poorer correlations between microarray and RNAseq data than high abundance genes. As expected, due to measurement and normalization differences, Agilent two-channel microarray and RNAseq data were poorly correlated (Spearman correlations coefficients of only ∼0.2). By examining the differentially expressed genes between tumor and normal samples we observed reasonable concordance in directionality between Agilent two-channel microarray and RNAseq data, although a small group of genes were found to have expression changes reported in opposite directions using these two technologies. Overall, RNAseq produces comparable results to microarray technologies in term of expression profiling. The RNAseq normalization methods RPKM and RSEM produce similar results on the gene level and reasonably concordant results on the exon level. Longer exons tended to have better concordance between the two normalization methods than shorter exons.

## Background

Gene expression analysis is essential for biomedical research. Expression profiling is the simultaneous measurement of the cellular concentration of different messenger RNAs. Microarrays have been the most popular high-throughput gene expression profiling technology for several decades. Recently, the introduction of RNAseq technology has had a revolutionary impact on the field of expression research. RNAseq refers to the use of next-generation sequencing (NGS) technologies to sequence cDNA in order to get information about a sample's RNA content. Compared to the microarray technology, the RNAseq method offers several distinct advantages. First, the detection range of RNAseq is not limited to a set of predetermined probes as with the microarray technology, so RNAseq is capable of identifying new genes. Second, the resolution of a microarray is limited to the gene level for most arrays and the exon level for specially designed exon arrays. On the other hand, RNAseq can detect expression at the gene, exon, transcript, and coding DNA sequence (CDS) levels. Finally and most importantly, RNAseq can detect structural variants such as alternative splicing and gene fusion. With the maturity of NGS technologies, the price of RNAseq has become comparable to microarrays. The competitive price and additional genomic information make RNAseq an attractive alternative technology for expression profiling. Some researchers have predicted the inevitable replacement of microarray by RNAseq [1], [2]. However, before this replacement can occur, we must understand the differences and similarities of these two technologies.

There are two standard microarray detection paradigms: one-channel and two-channel, also known as one-color and two-color detection. Two-channel microarrays are hybridized with cDNA from a pair of samples to be compared, with one sample labelled with fluorescent Cy3 at a fluorescence emission wavelength of 570 nm (green), and the other with Cy5 at a fluorescence emission wavelength of 670 nm (red). After labelling, the two samples are mixed and hybridized to one microarray. The microarray is then scanned for fluorescence intensity. Gene expression identified from a two channel microarray is often represented as a ratio of Cy3/Cy5. In one-channel microarrays, usually only Cy3 is used and only a single sample is hybridized to one microarray. Thus, two-channel microarrays do not truly reflect the abundance levels of a gene transcript but rather the relative abundance between two samples. A study [3] has shown good agreement with high correlation coefficients and high concordance of differentially expressed gene lists between one-color and two-color microarrays.

The Microarray Quality Control (MAQC) project has shown that there is a high level of intra-platform consistency across test sites and inter-platform concordance in terms of genes identified as differentially expressed by microarray methods [4]. Similar to these microarray tests, RNAseq data has been shown to estimate expression level with high reproducibility [5]. The majority of the previous studies showed moderate to good concordance rate between microarray and RNAseq results. However, few studies have focused on human gene expression consistency between RNAseq and microarray methods. The majority of existing studies have focused on non-human samples such as Candida parapsilolis [6], Candida albicans [5], fission yeast Schizosaccharomyces pombe [7], Drosophila melanogaster [8], Saccharomyces cerevisiae [9], Caenorhabditis elegans [10], mouse tissues [10], [11], and rat tissues [12]. A few studies [5], [13], [14] have performed comparisons using human samples or cell lines, but the sample sizes of those studies were very limited. A large scale, comprehensive analysis of RNAseq and microarray gene expression consistency using human data would benefit the research community and serve as guidance for future studies. A perfect dataset for conducting such a study is The Cancer Genome Atlas (TCGA) [15], [16].

TCGA is a massive, comprehensive, and collaborative project to catalogue genomic data for over 20 types of cancers by the National Cancer Institute (NCI), the National Human Genome Research Institute (NHGRI), and 27 institutes and centers of the National Institute of Health (NIH). Gene expression profiling is one of the major components of genomic data collected by TCGA. However, use of the gene expression profiling data in TCGA is complicated by the fact the gene expression data reported was obtained through a mixture of microarray and RNAseq technologies. Fortunately, many of TCGA's samples have had gene expression quantified using both technologies. This provided a good opportunity to study the repeatability and concordance of gene expression profiling between the two technologies on a large scale.

## Methods

### TCGA Data Description

From TCGA, we collected expression data on 4747 samples over 14 cancer types (TCGA public data until Dec 17, 2012). Out of 4747 samples, 2250 were expression profiled using Agilent G450A_07 arrays, 1269 were expression profiled using Affymetrix HT_U133 arrays, and 4064 were expression profiled using RNAseq. The overlap between Agilent and Affymetrix arrays was 1134 samples, the overlap between the Agilent array and RNAseq was 1662 samples, and the overlap between Affymetrix array and RNAseq was 699 samples. Table 1 describes the detailed sample distributions between technologies and cancer types. More detailed and updated overlap information was described at supplementary table 1.

**Table 1:** TCGA sample description.

Cancer Abbreviation	Cancer Name	Sample Type	RNASeq RPKM	RNASeq RSEM	Agilent	Affymetrix
BLCA	Bladder Urothelial Carcinoma	Tumor	56	122	0	0
		Normal	11	16	0	0
BRCA	Breast invasive carcinoma	Tumor	782	813	536	0
		Normal	102	106	63	0
COAD	Colon Adenocarcinoma	Tumor	192	192	155	0
		Normal	0	0	19	0
GBM	Glioblastoma Multiforme	Tumor	0	168	473	532
		Normal	0	0	10	10
HNSC	Head and Neck squamous cell carcinoma	Tumor	263	303	0	0
		Normal	31	37	0	0
KIRC	Kidney renal clear cell carcinoma	Tumor	471	469	72	0
		Normal	68	68	0	0
KIRP	Kidney renal papillary cell carcinoma	Tumor	16	63	16	0
		Normal	0	15	0	0
LGG	Brain Lower Grade Glioma	Tumor	0	174	27	0
		Normal	0	0	0	0
LIHC	Liver Hepatocellular Carcinoma	Tumor	17	17	0	0
		Normal	9	9	0	0
LUAD	Lung adenocarcinoma	Tumor	126	355	33	0
		Normal	37	57	0	0
LUSC	Lung Squamous Cell Carcinoma	Tumor	224	220	155	133
		Normal	17	17	0	0
OV	Ovarian serous cystadenocarcinoma	Tumor	420	266	561	586
		Normal	0	0	4	8
READ	Rectum adenocarcinoma	Tumor	72	71	69	0
		Normal	0	0	3	0
UCEC	Uterine Corpus Endometrioid Carcinoma	Tumor	335	333	54	0
		Normal	5	5	0	0

Level 3 released gene level expression data for microarray, gene and exon level expression data for RNAseq were downloaded for 14 cancers from TCGA. The data processing and quality control were done by Broad Institute's TCGA workgroup. For microarray data, gene level normalization was performed by using Robust Multi-array Average (RMA) [17] algorithm on GenePattern [18]. Agilent expression values were gene centred. The RNAseq gene expression level 3 data contains Reads per Kilobase per Million mapped reads (RPKM) [19], RNAseq by Expectation-Maximization (RSEM) [20] and read count. RPKM is the most widely used RNAseq normalization method, and is computed as follows: RPKM  = 10^9^(C/NL), where C is the number of reads mapped to the gene, N is the total number of reads mapped to all genes, and L is the length of the gene. An alternative form of RPKM is Fragments Per Kilobase of transcript per Million mapped reads (FPKM) [21]. FPKM is computed similarly to RPKM, except it accounts for the scenario in which only 1 end of a pair-end read is mapped. RSEM on the other hand is based on a generative probabilistic model of maximum expectation. The major difference between RPKM and RSEM is that RPKM's normalization factor is proportional to the mean length of a transcript in the transcriptome while RSEM is independent of the mean expressed transcript length. The more detail difference between RPKM and RSEM is described in a study by Li et al. [22], section 1.1.1.

Both RPKM and RSEM results were generated using SeqWare pipeline [23]. The reference gene transcript set was based on the HG19 UCSC gene standard track. For RPKM, alignment of raw data was done using BWA [24], and for RSEM the alignment of raw data was done using MapSplice [25]. RPKM values were computed using the formula described earlier, and RSEM values were computed using RSEM package [20]. The detailed description of each processing protocol can be found in the TCGA open access FTP download directories.

### Comparison between RNAseq and Microarray

Consistencies between the RNAseq and microarray data were tested using Spearman's correlation instead of Pearson's correlation due to two reasons: 1. RMA normalization uses log2 transformation for microarray data, for RNAseq data, log transformation is impractical due to the large number of zeroes that often are reported in this method. 2. Pearson's correlation is heavily influenced by outliers, and RNAseq data is heavily skewed. In addition to raw expression correlation, the directionality and agreement of the significantly differentially expressed gene list between the two technologies are also important measurements of concordance. Breast cancer is the only cancer type in TCGA that collected expression data using both RNAseq and microarray on 53 tumor-normal paired samples. Using breast cancer data, we identified significantly differentially expressed genes based on Benjamini-Hochberg adjusted p-value and fold-change between the tumor and normal samples using paired t-tests on the microarray data. Previous studies have shown that edgeR [26] performs best among the read count based methods [27], [28]. Thus, for RNAseq data differentially expressed genes were selected based on fold change and adjusted p-value generated by the edgeR package. Directionality and significance agreements were computed. The consistency of the fold-change computed from the microarray and RNAseq data was evaluated by comparing the four quartiles of expression value. Furthermore, results of the RPKM and RSEM normalization methods were compared on both the gene and exon level.

## Results

### Expression Value Distribution

**Figure 1:**
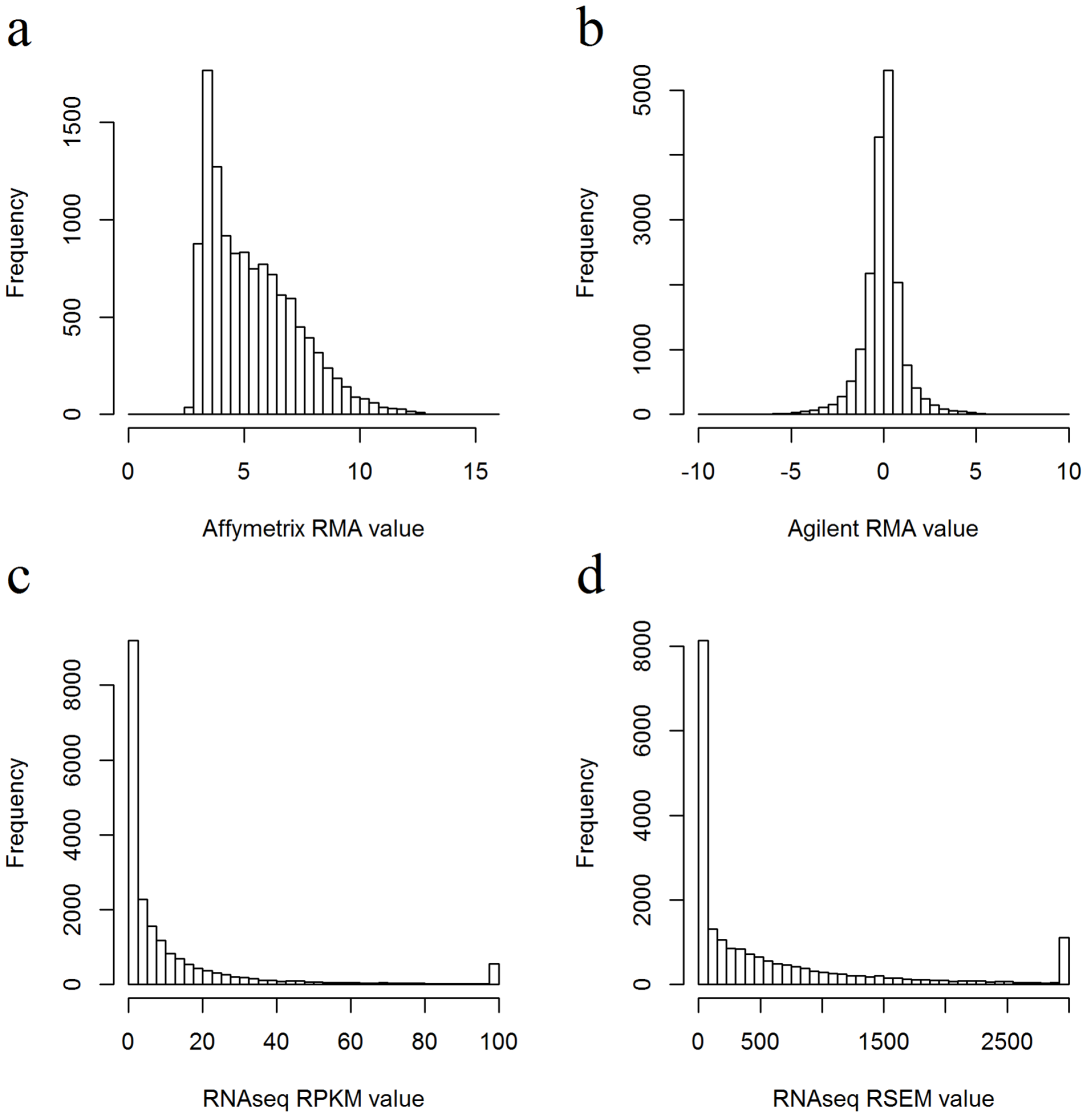
Expression value distributions of different quantification methods for the same 258 samples. For each method, each gene's expression value was represented by the median value from the 258 samples. a) Affymetrix microarray analysis followed by RMA normalization method. b) Agilent microarray analysis followed by RMA normalization method. c) RNAseq analysis followed by the RPKM normalization method, the last bar represents genes with RPKM over 100. d) RNAseq analysis followed by the RSEM normalization method, the last bar represents genes with RSEM over 3000.

Microarray and RNAseq methods are different in terms of the technology used to quantify gene expression. Microarray methods measure the intensity of fluorescence, which reflects the corresponding gene expression level, while RNAseq methods measure the read count, which also reflects the abundance of the gene product. The expression values of microarray data after RMA normalization are on a log2 scale. The count data of RNAseq, on the other hand, is not usually normalized using the quantile normalization method because a log-transformation does not provide a variance-stabilization of the data as it does for the (assumedly log-normally distributed) microarray data. However, in a study by Dillies et al. [27], quantile normalization has been evaluated in the context of RNA-seq data and shows no worse results than RPKM normalization. Figure 1 shows an example of expression level distributions across different platforms and normalization methods. The data generated by four methods from 258 same ovarian carcinoma samples were used in this comparison. For the Affymetrix microarray RMA normalization results, the range of expression was between 2.63 and 12.75 with a peak around 4 (Figure 1a); for the Agilent microarray RMA normalization, the range of expression was relatively symmetrically distributed between −7.66 and 7.61, with a peak around 0 (Figure 1b). The Agilent array had expression values across zero because expression from this array is computed as a ratio rather than as a raw intensity value. The expression values derived from RNAseq had a much wider distribution than that of the microarray methods (Figure 1c, 1d). For RPKM, the range was 0 to 3136.11, and for RSEM the range was 0 to 101988.11. Both RPKM and RSEM have large amounts of zero expression values (9.12% of RPKM  = 0, 12.58% of RSEM  = 0), which reflects non-expressed genes. For RPKM, out of 19990 total genes, the number of detected genes (RPKM >0) per sample was between 17330 and 18784 with the median equal to 18055. For RSEM, out of 20501 total genes, the number of detected genes (RSEM >0) per sample was between 16989 and 18725 with the median equal to 17835.

### Expression Concordance

The majority of cancer types in TCGA used the Agilent two-channel array for expression profiling. Three types of cancer, GBM, LUSC and OV (See Table 1 for definitions), used the Affymetrix one-channel array for expression profiling.

**Figure 2:**
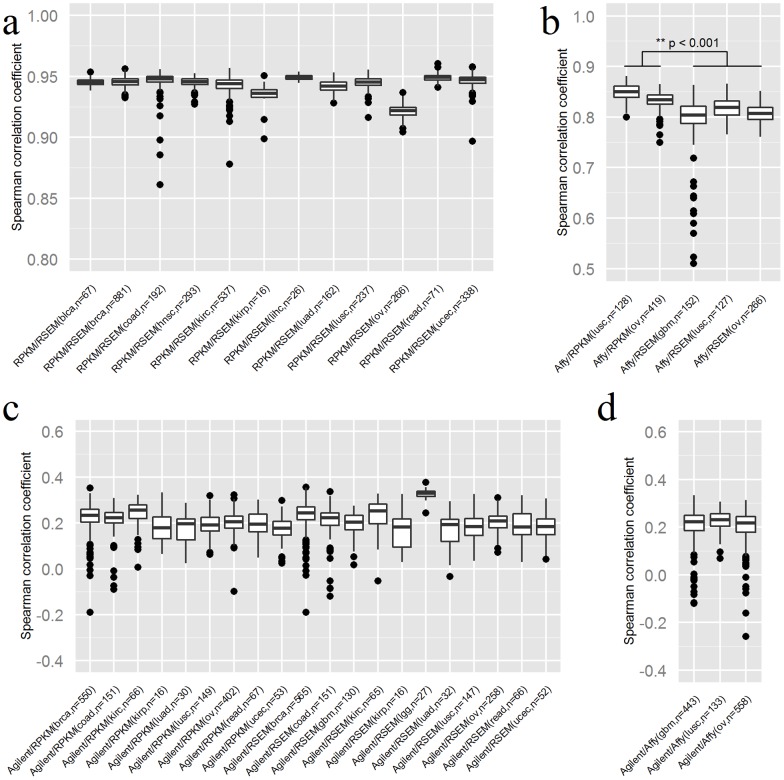
Spearman correlation coefficient analysis between different quantification methods. For each comparison, the samples from the tumor dataset that were analyzed by the corresponding methods were extracted. For each sample, the Spearman correlation coefficient of the expression values from those methods was calculated. a) The comparison between the RPKM method and the RSEM method. The Spearman correlation coefficients were as high as around 0.94. b) The comparison between the Affymetrix method and the RPKM/RSEM method. The Spearman correlation coefficients were around 0.8. c) The comparison between the Agilent method and the RPKM/RSEM method. Since the Agilent method generated a ratio value for each gene but the RNAseq methods generated an absolute expression value for each gene, the Spearman correlation coefficients between the Agilent method and the RNAseq methods were as low as ∼0.2. d) The comparison between the Agilent method and the Affymetrix method. Since the Affymetrix method also generated an absolute expression value for each gene, the Spearman correlations were also as low as ∼0.2.

We found high Spearman correlations between the RNAseq data normalized by the RPKM and RSEM methods across all types of cancers with median correlation coefficients range from 0.92 to 0.95 (Figure 2a). Good concordance between the Affymetrix one-channel array data and the RNAseq data was observed (Figure 2b). The median Spearman correlation coefficient for the Affymetrix microarray vs RPKM was from 0.83 to 0.85. For the Affymetrix microarray vs RSEM, the median Spearman correlation coefficient was from 0.80 to 0.82. A paired Wilcoxon test of Spearman correlation coefficients between the Affymetrix/RPKM and Affymetrix/RSEM comparisons from 383 common samples had a p-value of 4.3e-66 which indicates that the RPKM results were significantly more highly correlated to Affymetrix measurements than the RSEM results were.

We observed poor agreements between the Agilent two-channel array data and RNAseq data (Figure 2c). The median Spearman correlation coefficient for the Agilent microarray vs. RPKM for all cancers was from 0.18 to 0.26. For the Agilent microarray vs. RSEM, the median Spearman correlation coefficient was from 0.18 to 0.33. The low Spearman correlation between the two-channel microarray data and RNAseq data is a result of the normalization difference between ratio and non-ratio representations of the data. For two-channel microarray, the intensity of a gene is represented by the ratio of case vs control, which makes two-channel microarray data only comparable within a platform. Since RNAseq is a direct count measurement of gene transcript abundance, a low correlation in this case does not necessarily indicate low concordance between two-channel microarray data and RNAseq data. Poor agreement between the Agilent two-channel array data and the Affymetrix one-channel array data was also observed due to the same reason (Figure 2d).

### Differentially Expressed Genes Concordance

The primary purpose of gene expression profiling is to identify differentially expressed genes. TCGA has collected expression data on 53 paired tumor-normal breast cancer samples using both microarray and RNAseq methods. Significantly differentially expressed genes were identified using this paired breast cancer data. For a gene to be significantly differentially expressed between the tumor and normal samples, it has to satisfy two conditions: FDR adjusted p-value <0.01 and |Fold Change| >2. A standard paired t-test was used to compute the p-value for the microarray data. The Bioconductor package edgeR [26] was used to compute the p-values and fold changes for the RNAseq read count data downloaded from TCGA.

**Figure 3:**
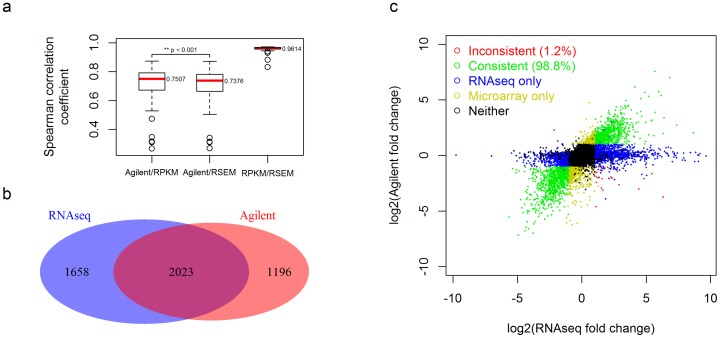
Differentially expressed gene concordance analysis using 53 paired tumor-normal breast cancer samples. a) The Spearman correlation coefficients of tumor/normal ratios between the Agilent method, the RPKM method and the RSEM method. b) Venn diagram summarizing the overlap between genes called as significantly differentially expressed (adjusted FDR less than 0.01 and fold-change larger than 2). The differentially expressed genes in Figure 3b were computed using commonly measured genes between microarray and RNAseq. c) Scatter plot of fold-change per gene as measured by the Agilent method and the RNAseq RPKM method. Genes identified as differentially expressed with consistent fold-change direction by both methods are plotted in green. Genes identified as differentially expressed with inconsistent fold change direction by both methods are plotted in red. Genes identified as differentially expressed by either RNAseq method or Agilent method are plotted in blue and yellow, respectively. Genes not identified as differentially expressed by either method are plotted in black. Only 1.2% genes identified as differentially expressed genes by both methods were inconsistent on the fold-change direction (red data).

When comparing the expression fold-changes of all genes, we observed generally good concordance between the microarray and RNAseq results (Figure 3a). Between the Agilent microarray and the RPKM method, the median Spearman correlation of the fold change was 0.75. Similarly, between the Agilent microarray and the RSEM method, the median Spearman correlation was 0.74. The median Spearman correlation of the fold change between the RPKM and RSEM normalization methods for RNAseq data was 0.96. A paired Wilcoxon test of the Spearman correlation coefficients between Agilent/RPKM and Agilent/RSEM comparisons was performed and the p-value was 1.2e-7 which indicates that the RPKM result was slightly but still significantly more similar to the microarray results than was the RSEM result.

There were 3219 genes identified as significantly differentially expressed by the microarray data and 3681 genes identified by the RNAseq data by edgeR. The overlap between the microarray and RNAseq results was 2023 genes (Figure 3b). Among these 2023 genes, there were only 24 genes with an inconsistent direction of change (consistency rate  = 0.988) (Figure 3c). The fold change and p-values of these 24 genes are reported in Table 2. From a biological point of view, besides the number of commonly differentially expressed genes, it is of interest to understand whether a biological interpretation of differentially expressed genes independently identified by each technique lead to the identification of the same deregulated pathways or not. To evaluate this, we performed pathway and functional analyses using Ingenuity Pathway Analysis (IPA). The results between genes identified by RNAseq and microarray showed some similarity but with enough difference to distinguish them clearly. This is understandable because there was a large non overlapped subset of significant genes identified by the two methods as shown in Figure 3b. The summary reports of IPA can be viewed in supplement table S2 and S3.

**Table 2:** Inconsistent significantly differentially expressed genes between microarray and RNAseq.

Gene	Microarray Log2 Fold Change	Microarray Adjusted Pvalue	RNAseq Log2 Fold Change	RNAseq Adjusted Pvalue
BMP8A	−1.05	8.43E-07	2.00	1.23E-32
CEACAM20	1.03	1.27E-09	−1.55	3.08E-03
CHGA	−1.66	1.46E-04	5.43	5.27E-13
COL9A1	−1.43	6.01E-04	2.76	8.04E-07
DCD	−3.74	1.99E-03	6.30	4.56E-10
GPM6A	−1.36	8.41E-04	1.56	3.13E-04
GRIA3	−1.53	1.26E-03	1.01	3.17E-03
GSTA3	−3.02	2.59E-07	4.41	9.71E-09
IGF2	−1.51	1.12E-04	1.16	1.84E-04
KLK11	−4.60	3.66E-04	1.10	1.88E-03
KRT16	−2.03	1.34E-03	2.34	1.48E-07
KRT6A	−2.93	3.51E-05	1.52	2.68E-03
LEMD1	−3.12	7.20E-04	1.63	7.73E-03
LGALS7	−1.94	3.25E-06	1.16	5.48E-03
LY6D	−2.63	5.45E-05	2.88	2.94E-06
MT1H	−1.23	4.69E-09	2.22	1.46E-06
PLAT	−1.85	1.02E-04	1.01	8.23E-03
PLP1	−2.42	1.03E-08	1.12	4.74E-03
PRSS12	−2.07	8.56E-04	1.56	1.52E-03
RLBP1	−1.05	1.34E-04	1.49	8.72E-03
SCN1A	−1.06	3.07E-04	4.99	3.59E-13
TNFSF11	2.29	3.40E-03	−2.16	1.17E-05
UCP1	−1.95	7.10E-06	2.24	2.55E-05
UGT2B4	−2.08	3.96E-04	4.81	1.25E-16

**Figure 4:**
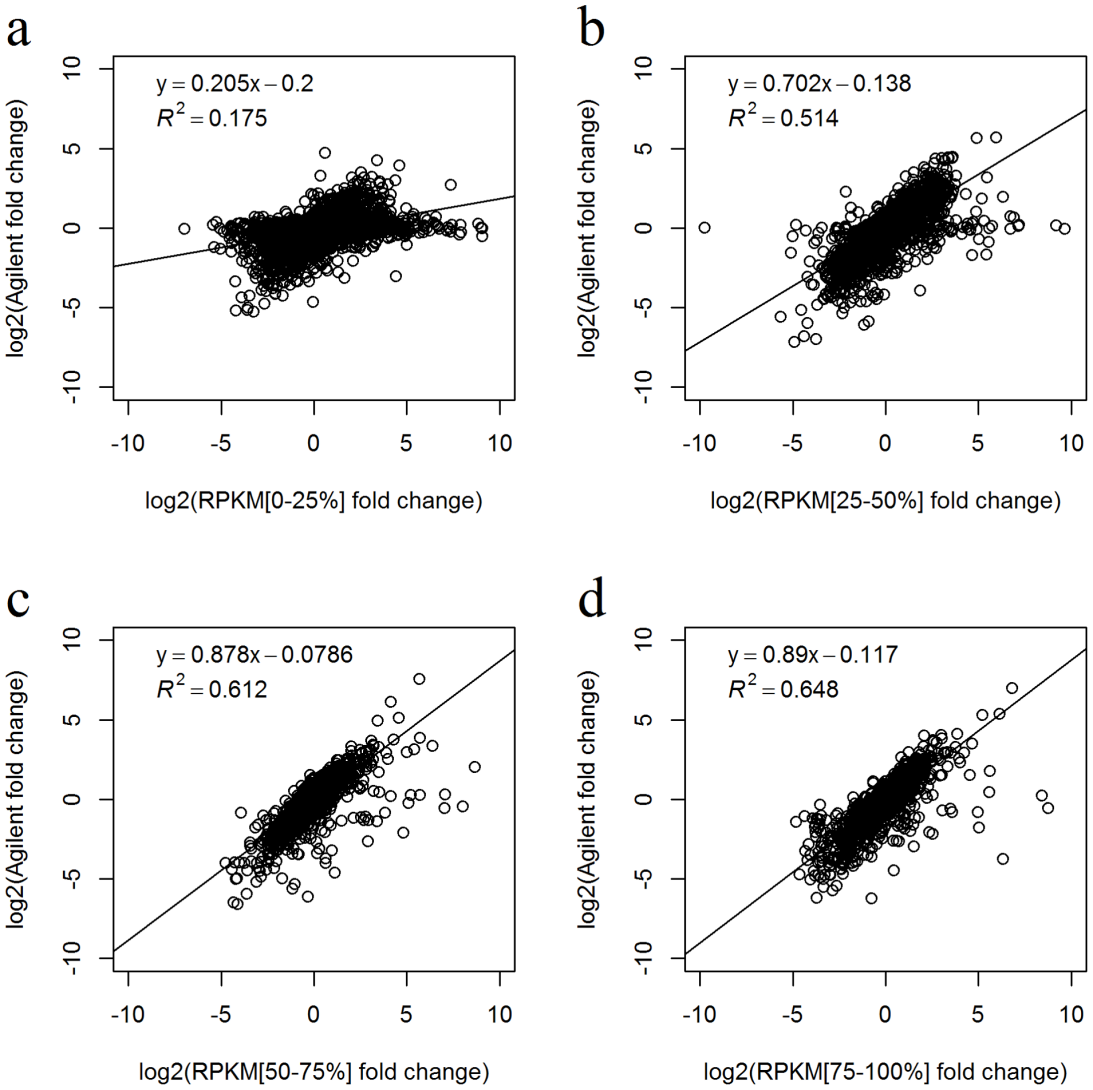
Fold-change consistency between the Agilent method and the RPKM method from 53 paired tumor-normal breast cancer samples. The common genes were divided into four groups based on their RNAseq expression value, and linear regression was performed to evaluate the fold-change consistency for each group. This indicates that the fold-change derived from genes with higher RNAseq expression was more concordant with the fold-change derived from microarray expression than the fold-change derived from genes with lower RNAseq expression.

The consistency of the expression fold-change between the microarray and RNAseq data was further evaluated by dividing the RNAseq expression values into four quartiles (Figure 4). A linear regression model was used to fit the fold change results within each expression quartile. A monotonically increasing pattern of consistency was observed as the gene expression level (as measured by RNAseq) increased. For the four quartiles of RPKM expression, the linear regression model's R^2^ equals 0.175, 0.514, 0.612 and 0.648 respectively. In the previous study conducted by Wang et al., the authors found poor concordance between raw high RNAseq expression and microarray expression. Our results contradict that study by showing that the concordance between microarray and RNAseq measurements of fold-change increases with higher expression level [1].

### RPKM and RSEM

**Figure 5:**
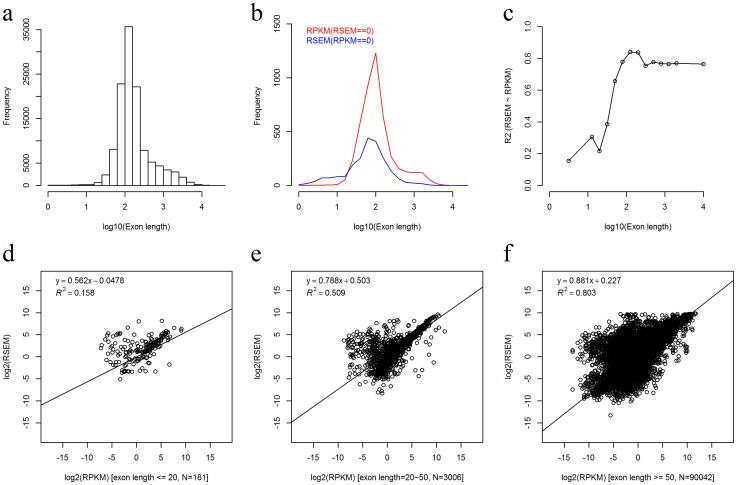
Exon expression consistency between the RPKM and RSEM normalization methods for RNAseq data. a) Exon length distribution from RNAseq data. Exons were divided into 23 groups based on log10 value of exon length. b) The length distribution of exons, blue indicates exons were detected by RSEM but not by RPKM, red indicates exons were detected by RPKM but not by RSEM. c) The 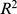 of linear regression between the RPKM and RSEM values in sub-groups defined by the exon length. The group intervals equalled to the group intervals in figure 5a, except the first five and the last five groups were merged respectively due to small exon count in those groups. Only the exons detected by both RPKM and RSEM methods were used. d-f) The detailed scatter plots of exon expression consistency in three groups divided by exon length of 1∼20, 21∼50, and >50 base pairs. Only the exons detected by both RPKM and RSEM methods were used. Figures c-f indicate that the exon expression consistency increases significantly with exon length until exon length is larger than about 50 base pairs.

RPKM/FPKM is currently the most popular method for normalizing RNAseq gene expression. The RSEM method has gained considerable popularity, evidenced by the recent adaptation of this method for all RNAseq data by TCGA. We have shown excellent agreement between the RPKM and RSEM methods at the gene level. To be more thorough, we also compared RPKM and RSEM results at the exon level. For RNAseq, the length of the exon plays a significant role in detectability. The median exon length was 138, but the mean exon length was 345, as this data was biased by very long exons (Figure 5a). The consistency between the RPKM and RSEM methods on the exon level is good but not as strong as on the gene level. Some exons were detected as expressed by one of the normalization methods but were not detected by the other, which suggests the level of disparity between RPKM and RSEM is quite strong for these exons (Figure 5b, d). We divided the exons into subcategories based on their length, (1–25, 26–50, >50 base pairs). Linear regression models were used to fit the exon expression data between the RPKM and RSEM methods at each length interval. The R^2^ of the linear regression shows that for very short exons the consistency between the RPKM and RSEM methods is poor compared to exons with longer length (Figure 5c, d, e, f). This directly reflects the fact that expression of short exons is harder to quantify through the sequencing method.

## Discussion

With RNAseq gradually taking over as the tool of choice for expression profiling, microarray technology is facing a tough battle to stay relevant. Microarray companies such as Affymetrix and Agilent have not abandoned microarray technology. Instead, more customizable and higher resolution arrays are being produced to compete for market share. These strategies will prolong the life of microarray technology, but they will not change their inevitable replacement by RNAseq technology.

Some researchers choose RNAseq over microarray without a thorough understanding of the differences between the methods. The microarray technology does still hold several advantages over RNAseq. One of the advantages is the lesser complexity required for analysis. Choosing the proper normalization method for a platform may depend on many variables. For microarray data, especially for the Affymetrix and Agilent platforms, one of the most acceptable normalization methods is RMA. To detect differentially expressed genes, a simple t-test is acceptable in most scenarios for microarray data. On the other hand, more complicated models have been introduced to deal with RNAseq's non-expressed genes such as negative binomial: DESeq [29], edgeR [26], baySeq [30], NBPseq [31], and Poisson distribution: TSPM [32], DEGseq [33]. There has been several studies [27] [28] [34] [35] attempting to compare different normalization and differential gene comparison methods for RNAseq data. In the study by Dillies et al. [27], through simulation, the authors found DESeq [29] and edgeR [26] are able to maintain a reasonable false-positive rate without any loss of power. In a separate study by Kvam et al., the authors recommended baySeq, DESeq and edgeR. Robles et al. suggested using a combination of multiple packages may overcome possible bias susceptibility of a given package to a particular dataset of interest [28]. In a most recent study by Soneson et al. the authors suggested an unique combination use of existing packages with LIMMA [36] which performed well under many conditions. But such approach requires at least 3 samples per condition to have sufficient power to detect any differentially expressed genes. The authors also pointed out that the non-parametric based SAMseq [37] was among the top performers. However, due to the fold change required for statistical significance by SAMseq was lower than for many other methods, the differentially expressed genes identified by SAMseq may potentially have less biological significance. Thus far, a consensus on the best approach for RNAseq data analysis has not been reached.

Microarray analysis is more time and cost-efficient than is RNAseq data analysis. The typical raw file size for microarray data ranges from 10 MB to 100 MB while the size of a raw RNAseq data file ranges from 5 GB to 10 GB with additional space required to perform data analysis. To identify a significantly differentially expressed gene from the raw data using a microarray will only take hours; for RNAseq, it will take days to weeks depending on the sample size. In summary, although the current cost to perform expression profiling using microarray and RNAseq is comparable, the cost for the analysis and storage of RNAseq data is significantly higher than for microarray data. However, with the advance of computing hardware and maturity of RNAseq analysis algorithms, we expect these advantages of microarrays to slowly diminish.

Even though microarray technology ultimately cannot compete with RNAseq for expression profiling, it is still very useful for genotyping purposes. The recently introduced exome genotyping arrays by Illumina and Affymetrix cost significantly less than exome sequencing. Illumina's Human Exome BeadChip only costs $45 per array compared to $500–1000 per exome sequencing. Even with the limitation of a pre-selected SNP list, the exome chip is preferable for large scale genome wide association studies (GWAS) due to the more manageable price.

With microarray technology slowly fading into history, the huge amount of microarray expression data collected by researchers over the last decade is still very valuable for data mining. The majority of the microarray data have been organized and stored in publicly available databases. The Gene Expression Omnibus (GEO) contains expression data on 848,178 samples across 2720 datasets, and ArrayExpress contains 988,372 assays across 34,148 experiments. Those microarray databases are still providing important support and information for researchers across the world every day.

Our results show that two-channel microarray expression data should not be directly compared to RNAseq expression data due to different detection schemes. One-channel microarray has very good concordance with RNAseq expression data. Reasonable agreement was observed between the significantly differentially expressed gene lists identified by two-channel microarray and RNAseq techniques; however, out of the significant genes identified by both RNAseq and two-channel microarray, about 1.2% genes had an opposite direction of the fold change. This inconsistency could be caused by random error, normalization differences or heterogeneity in samples. In the scenario of observing such inconsistency, it would be extremely difficult to decide which method to trust, especially when the difference is caused by sample heterogeneity. Thus, we recommend running multiple analysis methods such as bioconductor package LIMMA [36] for microarray, and read count based methods such as DESeq [29], edgeR [26], baySeq [30] to select reliable genes. If the discrepancy still cannot be resolved, wetlab method such as RT-PCR needs to be performed for validation. Even though such inconsistency percentage is very small, it does cause some concern and warrants further study. By comparing the RPKM and RSEM normalization methods, we found very good consistency at the gene level and good consistency rate at the exon level. However, the RPKM and RSEM normalization methods had poor consistency for short exons compared to long exons, partially due to the limitation of sequencing technology's ability to detect short exons and the resulting difficulty involved in correctly quantifying the expression of those short exons. Both absolute expression value from one-channel microarray data and fold-change value from two-channel microarray data show slightly more concordance to RPKM result than RSEM result, which may indicates that the RPKM method is more accurate than the RSEM method on gene expression estimation.

We have presented the largest microarray and RNAseq expression comparison study performed thus far. Given the large sample size, our results would be more definitive than previous studies on this subject. There is no denial that RNAseq is replacing microarray at a rapid pace. However there are still researchers who have yet to make transition from microarray to RNAseq. Our study provides definitive evidence that RNAseq can indeed replace microarray in term of expression analysis. Furthermore, our study also shows that results derived from microarray data are trust worthy. The huge amount of microarray data accumulated over last 10 years stored in repositories such as GEO and ArrayExpress can still serve as excellent data mining resources.

## Supporting Information

Table S1 **Shared samples between gene expression technologies in TCGA.**
(XLSX)

Table S2 **Inenuity pathway analysis summary for microarray results.**
(PDF)

Table S3 **Inenuity pathway analysis summary for RNAseq results.**
(PDF)
