# Supplementary material for: Large Scale Comparison of Gene Expression Levels by Microarrays and RNAseq Using TCGA Data
Source: PLoS One. 2013 Aug 20;8(8):e71462. doi: 10.1371/journal.pone.0071462 (PMC3748065; doi:10.1371/journal.pone.0071462)
Supplement: Table S2 — Inenuity pathway analysis summary for microarray results. (PDF) [file pone.0071462.s002.pdf]

Analysis Name: Figure3_Agilent_significant - 2013-06-25 01:15 PM
Analysis Creation Date: 2013-06-25
Build version: 220217
Content version: 16542223 (Release Date: 2013-05-13)

Analysis settings

View

Reference set: Ingenuity Knowledge Base (Genes Only)

Relationship to include: Direct and Indirect

Includes Endogenous Chemicals

Optional Analyses: My Pathways My List

Filter Summary:

Consider only molecules and/or relationships where

(species = Human OR Mouse) AND

(confidence = High (predicted) OR Experimentally Observed) AND

(tissues/cell lines = Other Colon Cancer Cell Lines OR LOX IMVI OR NCI-H332M OR Ovary OR Dendritic cells not otherwise specified OR Jurkat OR Immune cell lines not otherwise specified OR Leukemia Cell Lines not otherwise specified OR J774 OR Central memory cytotoxic T cells OR Thymus OR MOLT-4 OR HOP-92 OR Other Macrophage Cancer Cell Lines OR EKVX OR Lung Cancer Cell Lines not otherwise specified OR RPMI-8266 OR UACC-257 OR Other Lung Cancer Cell Lines OR Other Lymphoma Cell Lines OR Other Immune cell lines OR SK-MEL-28 OR Lung OR Bladder OR Skeletal Muscle OR Neutrophils OR Murine NKT cells OR RAW 264.7 OR Vd2 Gamma-delta T cells OR Other Myeloma Cell Lines OR Other T lymphocytes OR Vd1 Gamma-delta T cells OR KM-12 OR Naive helper T cells OR MALME-3M OR Macrophages OR NCI-H522 OR SK-MEL-2 OR HCC-2998 OR Salivary Gland OR HCT-116 OR BDCA-3+ dendritic cells OR Monocytes OR Central memory helper T cells OR SK-MEL-5 OR Kidney OR Other B lymphocytes OR CCRF-CEM OR Other Organ Systems OR THP-1 OR

(c) 2000-2013 Ingenuity Systems, Inc. All rights reserved.

Other Tissues and Primary Cells OR Plasmacytoid dendritic cells OR NK cells not otherwise specified OR NCI-H226 OR Retina OR Th1 cells OR Cytotoxic T cells OR Melanoma Cell Lines not otherwise specified OR Activated CD56dim NK cells OR Adipose OR K-562 OR Mature monocyte-derived dendritic cells OR Testis OR Myeloma Cell Lines not otherwise specified OR Small Intestine OR Heart OR Activated CD56bright NK cells OR Memory B cells OR Uterus OR Other Dendritic cells OR M14 OR Colon Cancer Cell Lines not otherwise specified OR Spleen OR Effector T cells OR HCT-15 OR Immature monocyte-derived dendritic cells OR Effector memory cytotoxic T cells OR Natural T-regulatory cells OR Cell Line not otherwise specified OR SR OR HT29 OR Th2 cells OR Epidermis OR Pancreas OR Activated Vd1 Gamma-delta T cells OR Other Melanoma Cell Lines OR A549-ATCC OR UACC-62 OR Effector memory RA+ cytotoxic T cells OR HL-60 OR Organ Systems not otherwise specified OR HOP-62 OR Other Immune cells OR SW-620 OR Other Leukemia Cell Lines OR BDCA-1+ dendritic cells OR Lymphoma Cell Lines not otherwise specified OR Immune cells not otherwise specified OR Mammary Gland OR Activated helper T cells OR Placenta OR Monocyte-derived macrophage OR Stomach OR Other Cell Line OR Liver OR NCI-H23 OR Prostate Gland OR CD56dim NK cells OR Effector memory helper T cells OR Macrophage Cancer Cell Lines not otherwise specified OR CD56bright NK cells OR Tissues and Primary Cells not otherwise specified OR T lymphocytes not otherwise specified OR Naive B cells OR Large Intestine OR COLO205 OR Activated Vd2 Gamma-delta T cells OR Cells not otherwise specified OR Other NK cells OR H460 OR Other Cells OR B lymphocytes not otherwise specified)
AND
(data sources = BIND OR BIOGRID OR Cognia OR DIP OR Gene Ontology (GO) OR Ingenuity Expert Findings OR Ingenuity ExpertAssist Findings OR INTACT OR Interactome studies OR MINT OR MIPS OR miRBase OR miRecords OR TarBase OR TargetScan Human)
Cutoff:
Fold Change = 2.000

Top Networks

ID	Associated Network Functions	Score
1	Cellular Assembly and Organization, Cancer, Reproductive System Disease	39
2	Cancer, Dermatological Diseases and Conditions, Developmental Disorder	36
3	Connective Tissue Disorders, Inflammatory Disease, Skeletal and Muscular Disorders	30
4	Connective Tissue Disorders, Dermatological Diseases and Conditions, Developmental Disorder	28
5	Endocrine System Development and Function, Small Molecule Biochemistry, Cellular Development	27

(c) 2000-2013 Ingenuity Systems, Inc. All rights reserved.

(c) 2000-2013 Ingenuity Systems, Inc. All rights reserved.

Top Bio Functions

Diseases and Disorders

Name	p-value	# Molecules
Cancer	7.78E-44 - 3.03E-04	589
Reproductive System Disease	7.78E-44 - 1.48E-04	248
Gastrointestinal Disease	1.33E-23 - 2.48E-04	288
Dermatological Diseases and Conditions	4.36E-17 - 3.13E-04	180
Connective Tissue Disorders	3.33E-14 - 2.43E-04	143

Molecular and Cellular Functions

Name	p-value	# Molecules
Cellular Movement	2.47E-22 - 3.24E-04	209
Cellular Growth and Proliferation	3.37E-15 - 2.72E-04	285
Lipid Metabolism	6.44E-13 - 2.64E-04	139
Molecular Transport	6.44E-13 - 3.07E-04	182
Small Molecule Biochemistry	6.44E-13 - 2.64E-04	149

Physiological System Development and Function

Name	p-value	# Molecules
Organismal Survival	2.12E-13 - 3.17E-05	251
Tissue Development	4.06E-12 - 1.79E-04	235
Behavior	8.59E-12 - 2.89E-04	112
Tissue Morphology	1.02E-10 - 2.91E-04	263
Cardiovascular System Development and Function	1.46E-10 - 3.00E-04	179

(c) 2000-2013 Ingenuity Systems, Inc. All rights reserved.

Top Canonical Pathways

Name	p-value	Ratio
Agranulocyte Adhesion and Diapedesis	9.2E-08	28/185 (0.151)
Granulocyte Adhesion and Diapedesis	4.03E-06	24/174 (0.138)
Hepatic Fibrosis / Hepatic Stellate Cell Activation	1.59E-05	20/142 (0.141)
Atherosclerosis Signaling	2.64E-05	18/133 (0.135)
Xenobiotic Metabolism Signaling	6.42E-05	30/289 (0.104)

Top Molecules

Fold Change up-regulated

Molecules	Exp. Value	Exp. Chart
COL11A1	↑7.573	
COL10A1	↑7.015	
GJB2	↑6.152	
MMP13	↑5.721	
PPAPDC1A	↑5.681	
MMP11	↑5.386	
CEACAM6	↑5.326	
CXCL11	↑5.116	
NEK2	↑4.948	
WISP1	↑4.501	

Fold Change down-regulated

(c) 2000-2013 Ingenuity Systems, Inc. All rights reserved.

INGENUITY® SYSTEMS

Molecules	Exp. Value	Exp. Chart
EDN3	↓-6.978	
NPY2R	↓-6.778	
FIGF	↓-6.573	
CA4	↓-6.472	
MUCL1	↓-6.222	
PCOLCE2	↓-6.165	
FABP7	↓-6.108	
FGFBP1	↓-6.066	
TNMD	↓-5.965	
CXCL2	↓-5.944	

Top Upstream Regulators

Upstream Regulator	p-value of overlap	Predicted Activation State
TNF	5.39E-23	
YY1	1.44E-18	
Cg	1.24E-17	Inhibited
FOXO1	1.77E-14	Activated
PPARG	3.20E-14	Inhibited

(c) 2000-2013 Ingenuity Systems, Inc. All rights reserved.

Top My Lists

Name	p-value	Ratio

Top My Pathways

Name	p-value	Ratio

Top Tox Lists

Name	p-value	Ratio
Cardiac Hypertrophy	6.64E-09	44/344 (0.128)
Acute Renal Failure Panel (Rat)	9.46E-07	14/60 (0.233)
Cardiac Fibrosis	1.07E-06	25/171 (0.146)
Xenobiotic Metabolism Signaling	3.67E-05	35/342 (0.102)
Hepatic Fibrosis	6.06E-05	15/95 (0.158)

(c) 2000-2013 Ingenuity Systems, Inc. All rights reserved.

INGENUITY® SYSTEMS

Top Tox Functions

Assays: Clinical Chemistry and Hematology

Name	p-value	# Molecules
Increased Levels of Alkaline Phosphatase	7.06E-03 - 3.78E-01	5
Increased Levels of ALT	3.19E-02 - 5.55E-01	2
Decreased Levels of Albumin	4.93E-02 - 3.97E-01	3
Increased Levels of CRP	9.63E-02 - 9.63E-02	1
Increased Levels of AST	1.16E-01 - 1.16E-01	2

Cardiotoxicity

Name	p-value	# Molecules
Cardiac Hypertrophy	4.82E-09 - 5.32E-01	40
Pulmonary Hypertension	2.59E-06 - 9.63E-02	12
Cardiac Fibrosis	2.27E-05 - 5.08E-01	24
Cardiac Congestive Cardiac Failure	4.82E-05 - 4.82E-05	16
Heart Failure	4.82E-05 - 1.83E-01	26

Hepatotoxicity

Name	p-value	# Molecules
Liver Hyperplasia/Hyperproliferation	2.12E-07 - 1.00E00	58
Hepatocellular Carcinoma	3.20E-05 - 1.00E00	42
Liver Steatosis	9.09E-04 - 1.41E-01	25
Liver Inflammation/Hepatitis	3.62E-03 - 3.33E-01	19
Liver Damage	4.41E-03 - 2.62E-01	17

(c) 2000-2013 Ingenuity Systems, Inc. All rights reserved.

Nephrotoxicity

Name	p-value	# Molecules
Renal Dysplasia	8.00E-04 - 4.93E-02	6
Kidney Failure	1.21E-03 - 3.71E-01	19
Renal Inflammation	2.14E-03 - 1.00E00	9
Renal Nephritis	2.14E-03 - 1.00E00	9
Renal Proliferation	2.53E-03 - 1.83E-01	21

(c) 2000-2013 Ingenuity Systems, Inc. All rights reserved.

INGENUITY® SYSTEMS
