# Supplementary material for: Large Scale Comparison of Gene Expression Levels by Microarrays and RNAseq Using TCGA Data
Source: PLoS One. 2013 Aug 20;8(8):e71462. doi: 10.1371/journal.pone.0071462 (PMC3748065; doi:10.1371/journal.pone.0071462)
Supplement: Table S3 — Inenuity pathway analysis summary for RNAseq results. (PDF) [file pone.0071462.s003.pdf]

Interactive pathway analysis of complex 'omics data

Analysis Name: Figure3_RNAseq_significant - 2013-06-25 01:13 PM
Analysis Creation Date: 2013-06-25
Build version: 220217
Content version: 16542223 (Release Date: 2013-05-13)

Analysis settings

View

Reference set: Ingenuity Knowledge Base (Genes Only)

Relationship to include: Direct and Indirect

Includes Endogenous Chemicals

Optional Analyses: My Pathways My List

Filter Summary:

Consider only molecules and/or relationships where

(species = Human OR Mouse) AND

(confidence = High (predicted) OR Experimentally Observed) AND

(tissues/cell lines = Other Colon Cancer Cell Lines OR LOX IMVI OR NCI-H332M OR Ovary OR Dendritic cells not otherwise specified OR Jurkat OR Immune cell lines not otherwise specified OR Leukemia Cell Lines not otherwise specified OR J774 OR Central memory cytotoxic T cells OR Thymus OR MOLT-4 OR HOP-92 OR Other Macrophage Cancer Cell Lines OR EKVX OR Lung Cancer Cell Lines not otherwise specified OR RPMI-8266 OR UACC-257 OR Other Lung Cancer Cell Lines OR Other Lymphoma Cell Lines OR Other Immune cell lines OR SK-MEL-28 OR Lung OR Bladder OR Skeletal Muscle OR Neutrophils OR Murine NKT cells OR RAW 264.7 OR Vd2 Gamma-delta T cells OR Other Myeloma Cell Lines OR Other T lymphocytes OR Vd1 Gamma-delta T cells OR KM-12 OR Naive helper T cells OR MALME-3M OR Macrophages OR NCI-H522 OR SK-MEL-2 OR HCC-2998 OR Salivary Gland OR HCT-116 OR BDCA-3+ dendritic cells OR Monocytes OR Central memory helper T cells OR SK-MEL-5 OR Kidney OR Other B lymphocytes OR CCRF-CEM OR Other Organ Systems OR THP-1 OR

(c) 2000-2013 Ingenuity Systems, Inc. All rights reserved.

Other Tissues and Primary Cells OR Plasmacytoid dendritic cells OR NK cells not otherwise specified OR NCI-H226 OR Retina OR Th1 cells OR Cytotoxic T cells OR Melanoma Cell Lines not otherwise specified OR Activated CD56dim NK cells OR Adipose OR K-562 OR Mature monocyte-derived dendritic cells OR Testis OR Myeloma Cell Lines not otherwise specified OR Small Intestine OR Heart OR Activated CD56bright NK cells OR Memory B cells OR Uterus OR Other Dendritic cells OR M14 OR Colon Cancer Cell Lines not otherwise specified OR Spleen OR Effector T cells OR HCT-15 OR Immature monocyte-derived dendritic cells OR Effector memory cytotoxic T cells OR Natural T-regulatory cells OR Cell Line not otherwise specified OR SR OR HT29 OR Th2 cells OR Epidermis OR Pancreas OR Activated Vd1 Gamma-delta T cells OR Other Melanoma Cell Lines OR A549-ATCC OR UACC-62 OR Effector memory RA+ cytotoxic T cells OR HL-60 OR Organ Systems not otherwise specified OR HOP-62 OR Other Immune cells OR SW-620 OR Other Leukemia Cell Lines OR BDCA-1+ dendritic cells OR Lymphoma Cell Lines not otherwise specified OR Immune cells not otherwise specified OR Mammary Gland OR Activated helper T cells OR Placenta OR Monocyte-derived macrophage OR Stomach OR Other Cell Line OR Liver OR NCI-H23 OR Prostate Gland OR CD56dim NK cells OR Effector memory helper T cells OR Macrophage Cancer Cell Lines not otherwise specified OR CD56bright NK cells OR Tissues and Primary Cells not otherwise specified OR T lymphocytes not otherwise specified OR Naive B cells OR Large Intestine OR COLO205 OR Activated Vd2 Gamma-delta T cells OR Cells not otherwise specified OR Other NK cells OR H460 OR Other Cells OR B lymphocytes not otherwise specified)
AND
(data sources = BIND OR BIOGRID OR Cognia OR DIP OR Gene Ontology (GO) OR Ingenuity Expert Findings OR Ingenuity ExpertAssist Findings OR INTACT OR Interactome studies OR MINT OR MIPS OR miRBase OR miRecords OR TarBase OR TargetScan Human)
Cutoff:
Fold Change = 2.000

Top Networks

ID	Associated Network Functions	Score
1	Organ Morphology, Skeletal and Muscular System Development and Function, Neurological Disease	37
2	Reproductive System Development and Function, Embryonic Development, Organ Development	32
3	Lipid Metabolism, Molecular Transport, Small Molecule Biochemistry	32
4	Cell-To-Cell Signaling and Interaction, Tissue Development, Cellular Function and Maintenance	32
5	Cell Cycle, Hepatic System Development and Function, Cancer	30

(c) 2000-2013 Ingenuity Systems, Inc. All rights reserved.

INGENUITY® SYSTEMS

(c) 2000-2013 Ingenuity Systems, Inc. All rights reserved.

Top Bio Functions

Diseases and Disorders

Name	p-value	# Molecules
Cancer	2.63E-35 - 3.87E-04	822
Reproductive System Disease	2.63E-35 - 2.42E-04	291
Respiratory Disease	2.24E-20 - 1.79E-04	496
Nutritional Disease	2.31E-17 - 1.42E-04	138
Neurological Disease	3.59E-16 - 4.26E-04	289

Molecular and Cellular Functions

Name	p-value	# Molecules
Lipid Metabolism	7.48E-13 - 1.97E-04	162
Molecular Transport	7.48E-13 - 4.28E-04	316
Small Molecule Biochemistry	7.48E-13 - 3.62E-04	219
Cell-To-Cell Signaling and Interaction	2.17E-12 - 4.01E-04	158
Cellular Movement	3.14E-11 - 3.50E-04	225

Physiological System Development and Function

Name	p-value	# Molecules
Behavior	1.16E-16 - 3.70E-04	165
Organ Morphology	1.88E-12 - 4.33E-04	190
Skeletal and Muscular System Development and Function	1.88E-12 - 4.33E-04	149
Nervous System Development and Function	2.17E-12 - 4.78E-04	218
Endocrine System Development and Function	2.27E-10 - 3.62E-04	99

(c) 2000-2013 Ingenuity Systems, Inc. All rights reserved.

INGENUITY® SYSTEMS

Top Canonical Pathways

Name	p-value	Ratio
Atherosclerosis Signaling	3.01E-08	28/133 (0.211)
Agranulocyte Adhesion and Diapedesis	3.31E-07	34/185 (0.184)
LXR/RXR Activation	4.43E-07	26/126 (0.206)
Granulocyte Adhesion and Diapedesis	6.12E-06	30/174 (0.172)
Serotonin Receptor Signaling	7.75E-05	10/33 (0.303)

Top Molecules

Fold Change up-regulated

Molecules	Exp. Value	Exp. Chart
S100A7A	↑9.653	
CPLX2	↑9.166	
KLHL1	↑9.048	
MAGEA12	↑8.829	
CARTPT	↑8.758	
CST4	↑8.649	
S100A7	↑8.418	
CASP14	↑8.021	
NEUROD4	↑7.890	
SSX1	↑7.808	

Fold Change down-regulated

(c) 2000-2013 Ingenuity Systems, Inc. All rights reserved.

INGENUITY® SYSTEMS

Molecules	Exp. Value	Exp. Chart
CSN2	↓-9.760	
MYOC	↓-5.682	
CFHR5	↓-5.462	
DLK1	↓-5.119	
ITLN1	↓-5.067	
KLK3	↓-5.026	
ANGPTL7	↓-4.931	
HBG1	↓-4.863	
LEP	↓-4.854	
SCGB1A1	↓-4.824	

Top Upstream Regulators

Upstream Regulator	p-value of overlap	Predicted Activation State
CTNNB1	4.43E-15	
CSF2	9.03E-14	Activated
YY1	2.73E-13	
HDAC1	8.78E-13	
IL1B	5.95E-12	

(c) 2000-2013 Ingenuity Systems, Inc. All rights reserved.

INGENUITY® SYSTEMS

Top My Lists

Name	p-value	Ratio

Top My Pathways

Name	p-value	Ratio

Top Tox Lists

Name	p-value	Ratio
LXR/RXR Activation	7.31E-07	26/124 (0.21)
Cardiac Hypertrophy	4.26E-06	49/344 (0.142)
Cardiac Fibrosis	1.52E-05	29/171 (0.17)
Acute Renal Failure Panel (Rat)	7.71E-05	14/60 (0.233)
Increases Renal Proliferation	3.45E-04	20/119 (0.168)

(c) 2000-2013 Ingenuity Systems, Inc. All rights reserved.

INGENUITY® SYSTEMS

Top Tox Functions

Assays: Clinical Chemistry and Hematology

Name	p-value	# Molecules
Increased Levels of Alkaline Phosphatase	1.50E-02 - 3.07E-01	7
Increased Levels of Potassium	1.62E-02 - 2.70E-01	5
Decreased Levels of Albumin	2.86E-02 - 2.02E-01	3
Increased Levels of ALT	6.50E-02 - 1.00E00	2
Increased Levels of Bilirubin	6.50E-02 - 6.50E-02	2

Cardiotoxicity

Name	p-value	# Molecules
Cardiac Arrythmia	5.19E-08 - 2.60E-01	34
Heart Failure	2.85E-07 - 1.40E-01	40
Cardiac Arteriopathy	5.85E-06 - 3.26E-01	43
Cardiac Congestive Cardiac Failure	5.09E-05 - 5.09E-05	20
Cardiac Hypertrophy	7.81E-05 - 2.02E-01	44

Hepatotoxicity

Name	p-value	# Molecules
Liver Hyperplasia/Hyperproliferation	8.36E-06 - 5.95E-01	71
Hepatocellular Carcinoma	3.25E-04 - 3.64E-01	52
Liver Failure	2.30E-03 - 6.69E-02	6
Liver Hyperbilirubinemia	5.27E-03 - 5.27E-03	2
Liver Inflammation/Hepatitis	6.26E-03 - 5.95E-01	31

(c) 2000-2013 Ingenuity Systems, Inc. All rights reserved.

INGENUITY SYSTEMS

Nephrotoxicity

Name	p-value	# Molecules
Kidney Failure	1.89E-04 - 3.98E-01	28
Renal Dysplasia	6.15E-04 - 7.26E-02	6
Renal Proliferation	1.61E-03 - 1.63E-01	26
Renal Inflammation	6.47E-03 - 1.00E00	18
Renal Nephritis	6.47E-03 - 1.00E00	18

(c) 2000-2013 Ingenuity Systems, Inc. All rights reserved.
